# Enhanced prediction and optimization of thin metal film optical properties using optimized ensemble learning models

**DOI:** 10.1038/s41598-025-27524-6

**Published:** 2025-12-10

**Authors:** Kevin Thomas, Amith Khandakar, Puvaneswaran Chelvanathan, Brahim Aissa, Mohammad Istiaque Hossain

**Affiliations:** 1 Department of Electrical Engineering, College of Engineering, Qatar University, Doha, Qatar; 2 Solar Energy Research Institute (SERI), National University of Malaysia (UKM), Bangi, Malaysia; 3 Qatar Environment and Energy Research Institute (QEERI), Hamad Bin Khalifa University (HBKU), Qatar Foundation, 34110 Doha, Qatar

**Keywords:** Metal thin films solar cells, Optical properties prediction, Multi-model stacking, Machine learning framework, Ensemble learning, %T, %R, %A prediction, Gradient boosting, XGBoost, Random forest, Data-driven modeling, Energy science and technology, Engineering, Materials science, Optics and photonics

## Abstract

Thin metal films are essential for expanding sensors, optoelectronic, and photovoltaic technologies. The intricate relationship between material composition, thickness, and production presents significant challenges in optimizing optical properties. The paper introduces an AI-driven framework for the simultaneous prediction and optimization of metal film optical characteristics, such as transmittance(%T), reflectance(%R), and absorptance(%A) using a dataset of 1320 experimentally measured samples across material films of gold, aluminum, nickel, tin, copper, and molybdenum over 200–2000 nm wavelength range. The input features to the model include wavelength and material type. Ensemble Models such as Random Forest, Gradient Boosting, XGBoost, and Extra Trees were trained and optimized through GridSearchCV with stratified K-fold cross-validation. A multitask learning model was also implemented to explore potential improvements from joint prediction. Among all models, the CatBoost Regressor demonstrated superior performance, achieving R^2^ = 0.99928, MAE = 0.21924, and MSE = 0.28203 on average across all outputs. To enhance interpretability, the feature importance analysis was employed, revealing that Material Type had a slightly more predictive influence than Wavelength. Additionally, material-specific error analysis identified challenging prediction zones tied to spectral extremes. The best performing machine learning model was deployed via a web-based GUI, enabling real-time prediction of thin-film optical properties. Overall, the proposed framework provides a scalable, interpretable, and deployable solution for AI-assisted material design and optical characterization. These findings accelerate thin metal film optimization by offering a reliable data-driven route for quick material property identification and enhancement through machine learning.

## Introduction

Modern technology heavily relies on thin metal films, which are used to improve sensing, photovoltaics, and optoelectronics^1^. Enhancing these films is critical for achieving better system efficiency due to the special optical characteristics of thin metallic coatings^2^. For achieving higher energy efficiency in solar photovoltaic (PV) converters, thin metallic coatings are essential^3^,^4^. Additionally, thin films can process light at nanoscale in optoelectronic devices, thereby enhancing LED functionality and display performance^5^. However, real-world applications face significant challenges, such as the delicate balance of optimum absorption of light with the lowest losses of energy^6^. These parameters could be altered by the thickness, composition, or production process.

Traditionally, theoretical calculations and experiments have been applied to the development and optimization of thin metal films. Simulations and empirical models have been used to evaluate and improve the optical characteristics of thin metal coatings. Correlations between material characteristics, film thickness, reflectance, transmittance, and absorptance are empirically determined in empirical modeling. These optical properties are often measured using methods like reflectometry and spectroscopic ellipsometry. Even though these techniques provide insightful information, they are time-consuming and labor-intensive, and they often call for iterative experimental attempts to provide the best findings.

Theoretical simulations have allowed for a more systematic understanding of light-matter interactions within metal films. Computational capabilities using the Transfer Matrix Method and Finite-Difference Time-Domain (FDTD) simulations allow researchers to model optical properties under different circumstances^7^. These simulations are very useful in studying complex geometries and multilayer systems. FDTD can reliably predict reflection and absorption spectra by solving Maxwell’s equations for certain film configurations. For example, prior studies have reported that FDTD and TMM-based optical simulations can require several minutes to hours per configuration, especially in inverse design settings or when fine mesh resolution and wide spectral ranges are considered^15–17^. These methods are generally less helpful for large-scale or high-dimensional parameter optimization due to the computational requirements. The alternative analytical methods, such as the Drude-Lorentz model or Fresnel equations, although easier to use, are usually limited in their ability to represent non-ideal or multi-material systems. Combining simulations and experimental data, hybrid techniques have become a viable avenue. Applications for these materials are becoming increasingly complex. Thus demanding the discovery and optimization of material properties that can be executed more rapidly and efficiently^8^,^9^. Machine learning (ML) has emerged as a paradigm shift in materials science, offering immense potential for data-driven optimization and predictive modeling. ML models can find patterns and correlations in datasets that may not be obvious through conventional techniques^10^.

Machine learning offers a powerful framework for hypothesis generation and experimental design, which reduces the number of iterations required to reach optimal configurations. In the case of thin metal film, it enables a faster discovery of materials and can be potentially used to revolutionize optical property predictions. Through examining sophisticated interactions between the composition of materials, thickness, and optical performance, ML suggest the best combinations that can fulfill a required optical application. Moreover, the robustness and adaptability of ML in treating complex material systems are enhanced by its ability to incorporate diverse data sources, such as computer simulations, experimental observations, and information gleaned from the literature^11^.

Recent advances in artificial intelligence have made significant progress in developing the optical properties of thin films. Oktay et al. predicted reflectance in Ge-based anti-reflection coatings with ensemble tree methods and deep neural networks^12^. On the same note, Wang et al. created a single deep neural network that was used to predict transmittance spectra of plasmonic nanostructures, but their model was isolated to transmittance and therefore used simulated data primarily during the training phase^13^. Although XGBoost ensemble model has been used by Mahani et al.^14^ to make predictions about the reflectance characteristics of Bragg gratings, it was applied only on one reflectance peak. Swe and Noh^15^ suggested an inverse design method using a tandem neural network in a multilayered manner, focusing mainly on reflectance minimization, with absorptance modeled implicitly. Although Ahmed et al. provided successful experimental validation for absorption optimization using inverse design and deep learning techniques, their study omitted the prediction or optimization of reflectance and transmittance properties^16^. Traditional design methods related to inverse design and physics-awareness were also brought to recent works in neighboring areas. Wang, et. al. presented a prior knowledge-informed inverse design of a full-stokes metasurface imager based on a non-interleaved shared-aperture metalens with high degree of accuracy and compactness of complex-system designs^17^. Elsewhere, Zhang et al. applied Multiphysics simulation and hybrid mode electromagnetic simulation used to optimize a 4-inch MPCVD reactor, to improve plasma stability and energy efficiency for large-scale diamond thin film deposition^18^.

Recent studies in related areas have also further demonstrated the feasibility of advanced AI techniques for predicting physicochemical properties. In particular, to calculate the heat capacity of deep eutectic solvents, Convolutional Neural Networks (CNN), Extreme Learning Machines (ELM), and Long Short-Term Memory (LSTM) networks were used, achieving remarkable accuracy^19^. Multigene genetic programming (MGGP) has also been successfully applied to estimating the solubility of CO_2_-N_2_ gas mixtures in brine in order to provide interpretable, high-performing models that could be applied in real-world carbon capture applications^20^. A flexible, interpretable alternative to ANN-based models of downhole mud viscosity under extreme temperature and pressure was offered in drilling fluid research, with Multivariate Adaptive Regression Splines (MARS) modeling downhole mud viscosity^21^. Such research represents the value of combining physical constraints and design automation as factors that can accomplish a breakthrough in performance and scale.

Most prior machine learning approaches in thin-film optics have been limited to single-output predictions, typically focusing on either transmittance or reflectance. This fragmented approach overlooks the fundamental physical interdependence between transmittance, reflectance, and absorptance, which are bound by the energy conservation law (%T + %R + %A = 100%). Models that ignore this relationship may generate predictions that are physically implausible or internally inconsistent. In contrast, a multi-output machine learning framework can jointly learn all three optical properties, ensuring coherent predictions that better reflect physical laws while dramatically reducing computational time. This joint modeling strategy not only improves predictive accuracy but also enhances scalability and robustness, enabling faster and more efficient material optimization pipelines.

Nonetheless, the introduction of ML has its own challenges, especially in this context. Data quality is often constrained by measurement noise, sample preparation variability, and limited experimental datasets, especially for newer material systems. They create an element of uncertainty in learning. Moreover, high-capacity models trained over a sparse data are at risk of overfitting, especially when cross-validated in multi-output. To mitigate these issues, ML models based on cross-validation, including domain knowledge in preprocessing, and physics-based penalization to regularize learning, can be combined. The combination of these measures enhances generalizability and makes prediction variance smaller.

While simulations remain foundational for understanding light matter interactions, ML-based approaches serve as an efficient substitute that enables rapid prototyping, optimization, and design exploration. Rather than replacing simulations, ML models can complement them by learning complex relationships from empirical data and reducing dependence on iterative numerical solvers, particularly in high-throughput screening tasks or inverse design. In view of these difficulties, herein, a multi-output machine learning framework is presented that is able to simultaneously predict transmittance, reflectance, and absorptance of different thin film metals.

This paper makes the following main contributions:

- Development of a high-quality experimental dataset across seven metal types over a 200–2000 nm spectral range.
- Implementation of a machine learning model to jointly predict %T, %R, and %A while respecting physical interdependence.
- Incorporation of uncertainty quantification**,** feature importance analysis, and material-wise residual evaluation for interpretability and robustness.
- Deployment of a real-time, user-friendly web application that integrates the proposed model to facilitate material design and optimization in practical settings

## Methodology

**Fig. 1:**
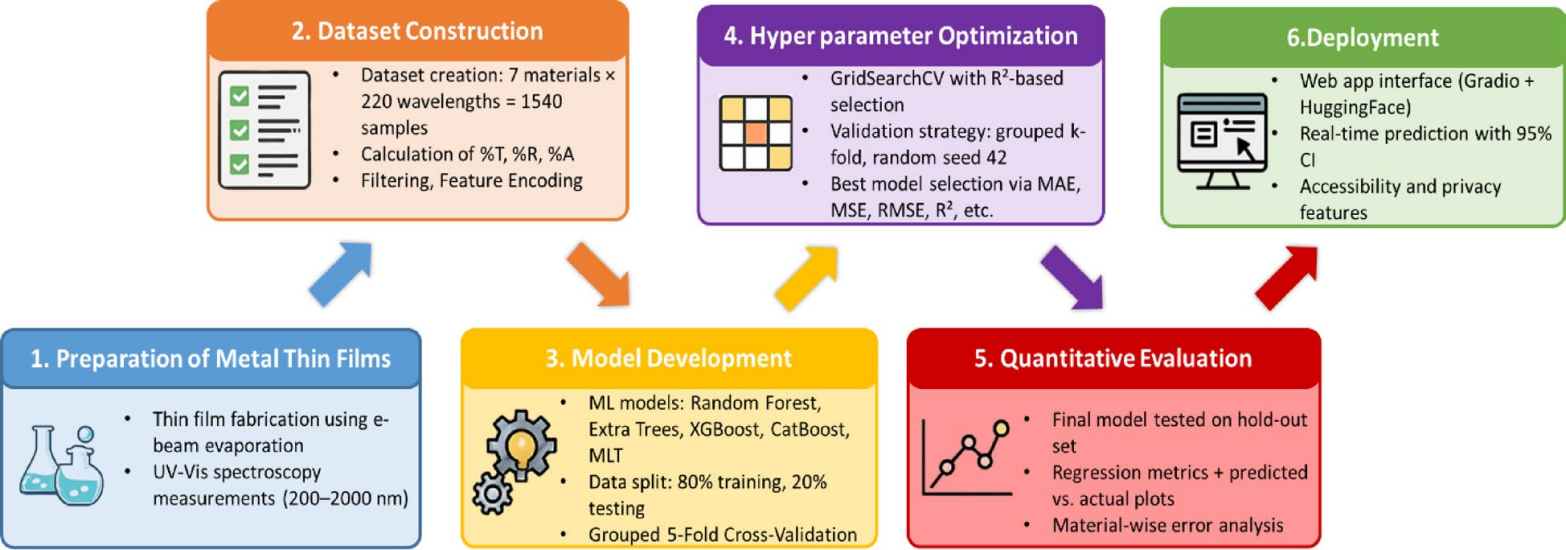
Methodology.

Our study presents the complete methodology as seen in Fig. 1 to ensure the reproducibility of results with high accuracy. The experimental workflow begins with the fabrication of thin metal films using electron-beam (e-beam) evaporation, conducted under tightly controlled conditions. High-purity metal sources (99.9995%) were used, with a consistent deposition rate of 1 Å/s and a carefully standardized substrate preparation protocol. The target thickness of 100 nm was consistently maintained, ensuring uniformity across all samples. Furthermore, the optical properties such as transmittance, reflectance, and absorptance were measured using ultraviolet–visible (UV–Vis) spectroscopy within a broad spectral range (200–2000 nm).

Additionally, the study provides a significant approach to machine learning model development, from dataset preparation to algorithm selection. A variety of predictive models were explored, including Random Forest, Gradient Boosting, XGBoost, CatBoost, Extra trees, and Multi Task Learning. Instead of proposing a novel model, we optimized existing machine learning models using GridSearchCV. Hyperparameters, including the number of estimators, maximum tree depth, learning rates, and minimum samples split, were systematically tuned. The best model was selected based on the highest R^2^ score, Mean Absolute Error (MAE), Mean Squared Error (MSE), MAPE, Adjusted R^2^ score, etc., across the validation set. An interactive web-based platform has been developed to integrate the predictive model. This tool serves as a practical validation framework, allowing researchers to compare their experimental findings with model-generated predictions.

### Preparation of metal films

#### Material preparation

Thin metallic films of various materials, including gold (Au), aluminum (Al), nickel (Ni), tin (Sn), copper (Cu), and molybdenum (Mo), were deposited onto soda lime glass substrates (dimensions 1″ × 3″) using e-beam evaporation. The deposition process was carried out at room temperature, maintaining a controlled rate of 1 Å/s without oxygen introduction. To ensure precision, the deposition rate was managed through automated software, which accounted for parameters such as tooling factors, material density, and Z-ratio to achieve a target thickness of 100 nm for each film. The optical properties of the resulting films were evaluated using ultraviolet–visible (UV–Vis) spectroscopy. Initial trials included fine-tuning the thickness of each layer to optimize optical performance. The e-beam evaporation system used, a Denton Vacuum Explorer™, allowed for the sequential deposition of multiple layers in a single vacuum cycle, preserving the structural integrity of the films. Prior to deposition, the glass substrates underwent thorough cleaning using a sequence of solvents, deionized water, acetone, and isopropanol, followed by drying under an inert nitrogen stream. The metallic evaporation pellets, sourced from Kurt J. Lesker, boasted a high purity level of 99.9995%, ensuring the quality of the deposited films. This meticulous approach highlights the importance of precision and cleanliness in thin-film fabrication for achieving desired material properties.

#### Optical properties of metal thin films

The optical properties of the films were studied using UV–Vis spectroscopy (Perkin ElmerTM) for the wavelength range of 200–2000 nm. Absorptance spectra were calculated using Eq. 1:

1 $$A\left( \% \right) = 100 - \left( {T + R} \right)$$

where A is the absorptance, T is the transmittance, and R is the reflectance.

**Fig. 2:**
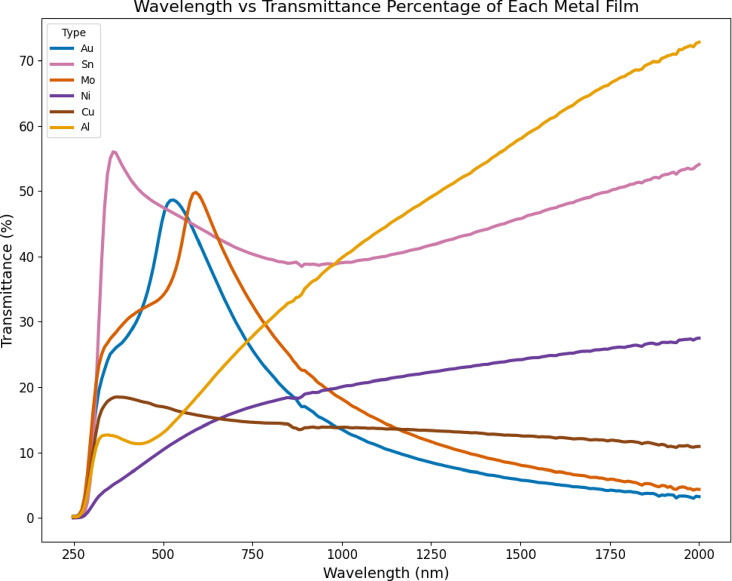
Transmittance of metal films from 200 to 2000 nm wavelength.

Spectra of transmission, reflectance, and absorptance show a distinct difference between all films developed with thicknesses of 100 nm for wavelengths between 200 and 2000 nm. Metallic characteristics therefore, provide clear criteria for further comprehension. The transmittance spectra of the thin metallic films are shown in Fig. 2 over a wide spectral range of 200–2000 nm. Transmittance is significantly low for metallic coatings because of their intrinsic reflectivity and light absorption characteristics. Figure 2 demonstrates how the transmittance characteristics of each metal cause it to behave very differently. Al exhibits the highest transmittance, starting near 10% in the UV and increasing almost linearly to exceed 70% beyond 1800 nm, indicating low absorption in the near-infrared. Ni and Cu maintain low transmittance (< 30%) across the entire range, consistent with strong attenuation. Au shows a pronounced peak of ~ 48% near 550 nm before declining at longer wavelengths, while Sn reaches a maximum of ~ 55% near 350 nm, followed by a gradual increase after 1000 nm. Mo exhibits a clear peak ~ 50% around 600 nm before decreasing steadily.

**Fig. 3:**
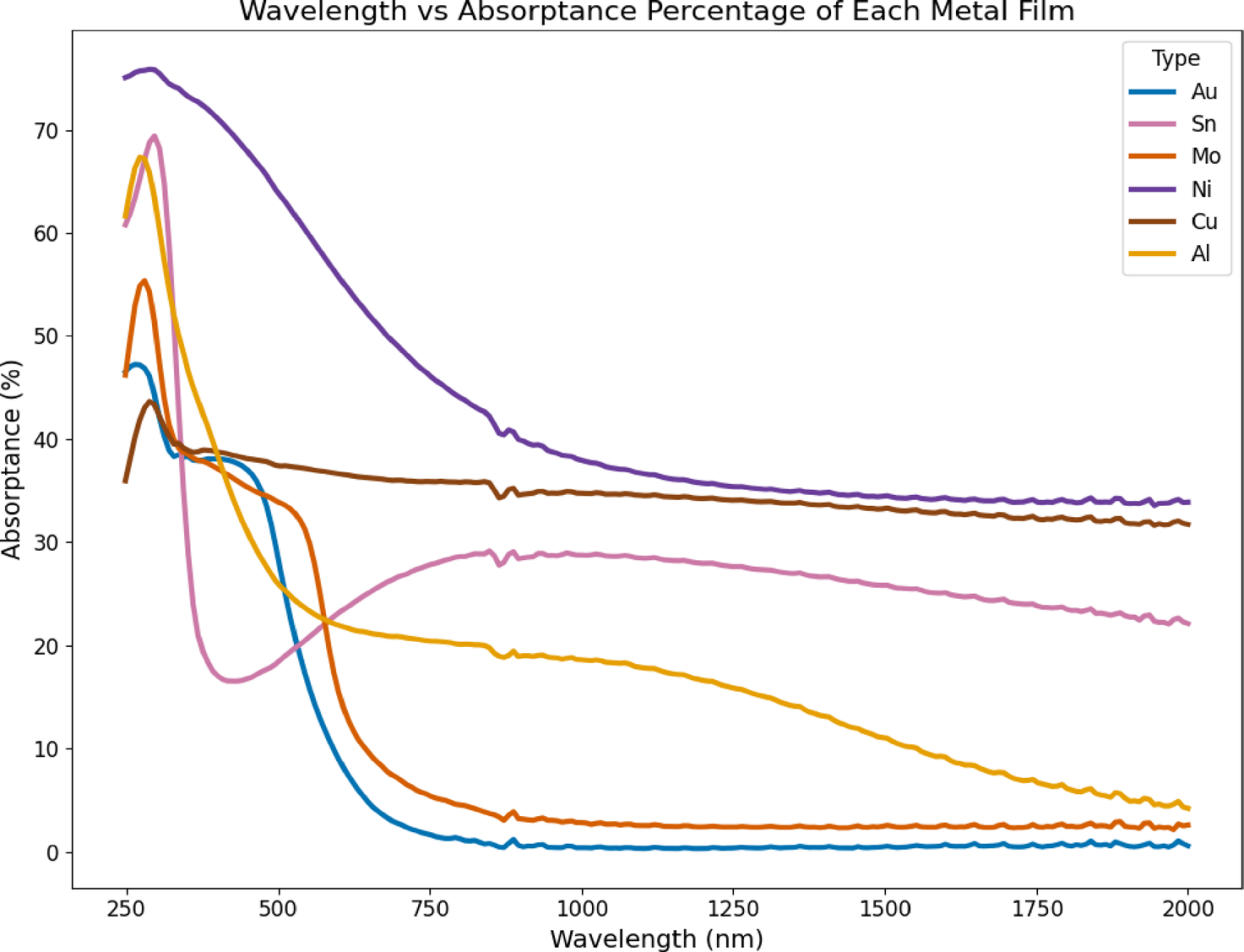
Absorptance of metal films from 200 to 2000 nm wavelength.

The absorptance of each material within the wavelength range is highlighted by the absorptance curve in Fig. 3. Metallic films show high absorptance in the UV region (< 400 nm), which generally decreases with increasing wavelength, although the decline rate varies per metal. The materials’ optical constants and electronic structure determine the rate. Ni shows the highest absorptance across most of the range, starting near 78% in the UV and gradually declining to ~ 33% at 2000 nm. Cu also maintains high absorptance ~ 65% in the UV to ~ 32% at 2000 nm. Al exhibits strong UV absorptance ~ 68% that falls sharply beyond 400 nm, dropping below 5% past 1500 nm, consistent with its high reflectivity. Mo and Au show moderate UV absorptance ~ 50–55% that declines steadily toward the infrared, while Sn starts high ~ 69% in the UV, drops sharply to ~ 18% around 450 nm, and then slowly rises beyond 1000 nm.

**Fig. 4:**
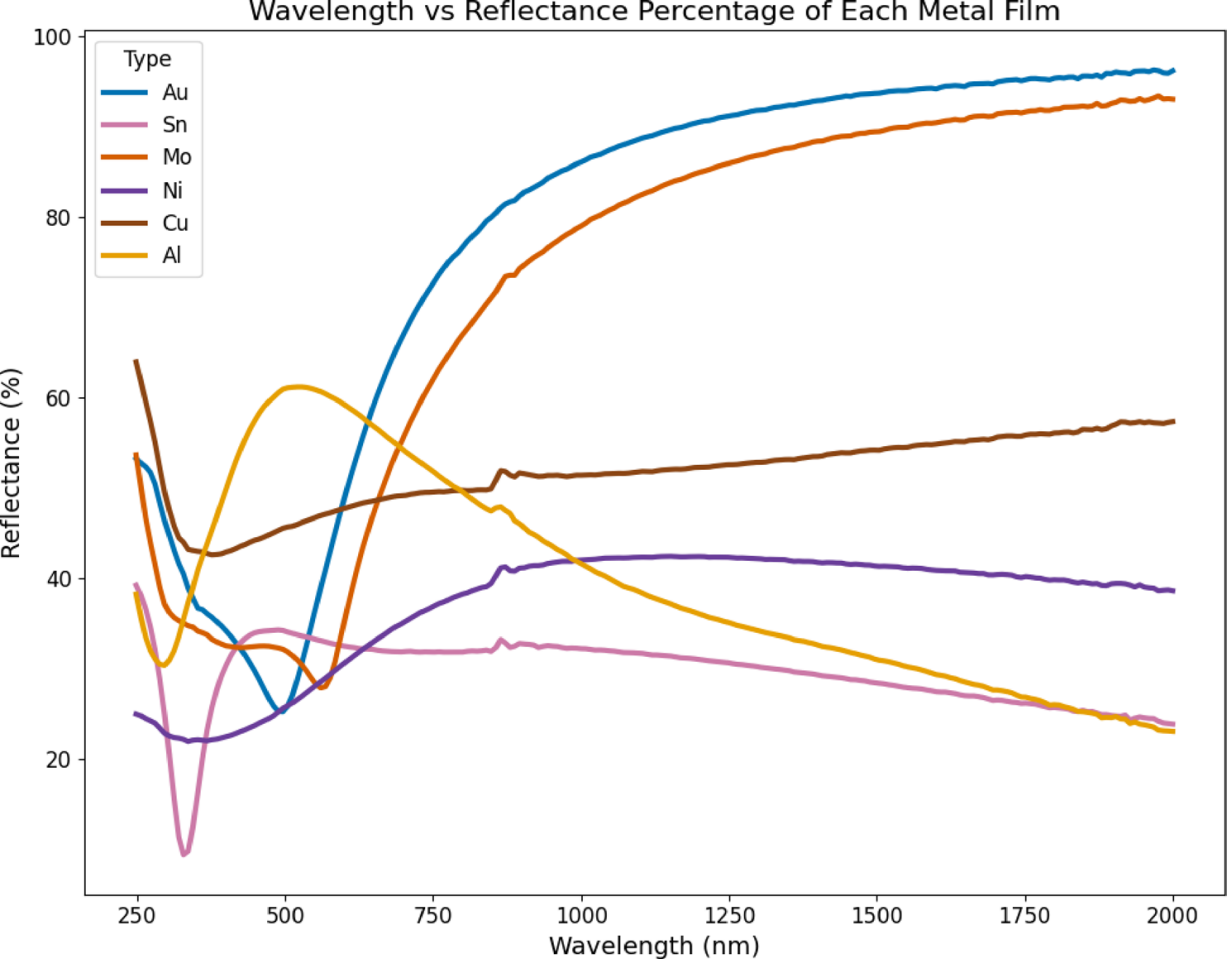
Reflectance of metal films from 200 to 2000 nm wavelength.

The individual metallic sheets reflections are described by reflectance spectra in Fig. 4. The reflectance spectra are highest for Al, starting above 70% in the visible–infrared range, and for Cu, which maintains ~ 50–55% in the visible to near-infrared region. Au exhibits strong reflectance in the infrared (> 50%), but reduced reflectance in the visible due to its plasmonic absorption, giving rise to its characteristic golden hue. Sn and Mo have lower overall reflectance, while Ni reflects the least, consistent with its high absorptance across the spectrum.

### Dataset preparation

This study’s dataset represents the experimentally measured optical properties of seven material classes: Gold (Au), Tin (Sn), Molybdenum (Mo), Nickel (Ni), Copper (Cu), and Aluminum (Al). Each material was measured across 220 unique wavelength points uniformly sampled between 200 and 2000 nm, resulting in a total of 1320 samples. The dataset also makes certain that material characteristics and optical performance are accurately represented to provide a rigorous description of this study. The following characteristics are included in the dataset as features:

- Wavelength: The light wavelength (measured in nanometers) at which the optical characteristics of the metal films are assessed.
- Material Type: The type of metal that was used to create the thin layer.

The following metrics are outputs to assess Optical Performance in the dataset:

- Transmittance Percentage (%T): The proportion of light that passes through the metal thin film
- Absorptance Percentage (%A): The proportion of light that the metal thin film absorbs.
- Reflectance Percentage (%R): The proportion of light that the metal thin film reflects.

All samples were screened for completeness. The dataset was confirmed to have no missing values across any of the features or target variables. The dataset was stratified by the type of material into 80% training set and 20% independent test set. The choice of an 80/20 split is consistent with standard machine learning practices that ensure sufficient data for both training and generalization assessment. In order to further consider and evaluate the generalizability of the model and to avoid over-fitting, a grouped fivefold Cross-Validation approach was used. This guarantees that all the material classes have a representative sample in individual folds and they offer stronger performance metrics and confidence intervals. The stratified splitting and cross-validation help to strengthen the validity of the results given.

**Fig. 5:**
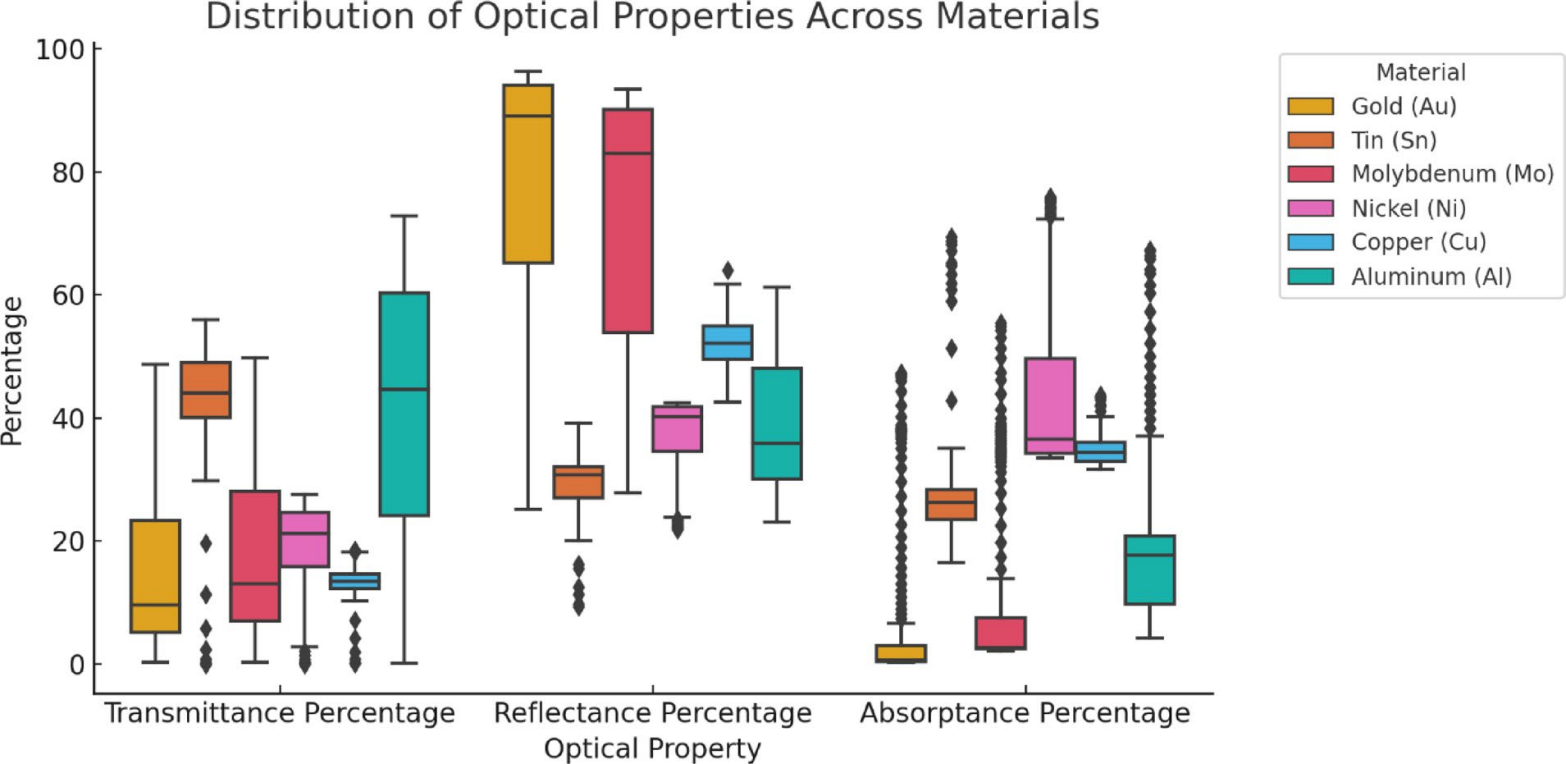
Boxplot based visualization of the distribution of optical properties.

Figure 5 presents a boxplot-based visualization of the distribution of transmittance, reflectance, and absorptance values across different material types. The plot reveals distinct differences in optical behavior across materials. Nickel exhibits the highest and most variable absorptance, while Aluminum shows consistently low absorptance but a wide range of transmittance values. Gold and Molybdenum demonstrate highly consistent reflectance patterns, whereas Tin displays moderate variability across all three properties. These variations highlight the dataset’s ability to capture nuanced and material-specific optical property relationships, making it well-suited for modeling and comparative analysis. This diversity confirms the suitability of the dataset for capturing complex material optical property relationships.

To address the concerns of experimental and predictive uncertainties in a very detailed and thorough manner, several layers of uncertainty analysis were introduced in this research. On the experimental front, the errors were measured on film thickness during the experimental run and the noise in the spectrometer (0.25%%) in calibrating the instrument itself. These experimentally observed variations were then propagated using a Monte Carlo-style perturbation approach, driven by actual measurement deviations rather than synthetic simulations. To measure the stability of the model performance, bootstrap resampling (n = 1000) with the training set was performed; confidence intervals (95%) of the main evaluation criteria, R^2^ and MAE, were measured. The machine learning models always exhibited a narrow band of confidence, which shows it is very reliable. Moreover, the web application under implementation will contain prediction intervals (95%) of its results i.e., transmittance, reflectance, and absorptance, and hence uncertainty in real-time prediction to the end consumer. Additionally, sensitivity analysis using feature perturbation indices indicated that material type contributes more significantly to output variance compared to wavelength, while intrinsic optical constants behave as expected within domain knowledge. Taken together, these analyses show that the framework suggested here is not only correct in ideal situations but also robust, interpretable, and uncertainty-aware, making it suitable for real-world optical design and optimization.

### Models for machine learning

#### Model selection

A broad range of studies have been conducted on machine learning approaches to predict optical attributes in thin films and materials, as reported in the literature. Different algorithms, including Decision tree, Deep Neural Network, and Tandem neural network, have been used as illustrated in Table 1. But these models tend to have shortcomings, e.g., they are not interpretable, sensitive to hyperparameters, or prone to overfitting on small data. Conversely, ensemble tree-based techniques (e.g. Random Forest, XGBoost, Extra Trees, and CatBoost) represent a more scalable option, which can be suited in dense and nonlinear cases such as the prediction of thin-film optical properties. These models can substantively represent nonlinear dynamics among wavelength, material composition, and film structure without the need for large preprocessing or feature creation. They naturally have a lower tendency to overfit because of regularization approaches such as bagging and boosting, and they accept heterogeneous input forms without many transformations. Moreover, ensemble models have an inbuilt interpretability in the form of feature importance analysis to allow the expert to detect how physical parameters affect the optical properties. Importantly, they can easily handle multi output regression, thereby predicting transmittance, reflectance, and absorptance all at once. Ensemble methods are also efficient computationally, compared to deep learning models and, these are more convenient when it comes to implementation in real-time systems. All these benefits make up the grounds to choose ensemble tree-based models as the core part of the predictive framework in this work.

**Table 1:** Overview of the experimental and machine learning studies for optical property prediction of thin metal films.

Study	Model(s)	Predicted outputs	Data source	Performance	Limitations
Oktay et al.^12^	Decision Trees, RF, DL, Neural Network	Reflectance	Simulated (FDTD)	DT MSE ≈ 6.9 × 10⁻⁷, DL MSE ≈ 4.0 × 10⁻^3^	Single-output; no experimental validation; DL underperformed
Ahmed et al.^16^	Deep Neural Network (inverse)	Absorptance	Simulated + 1 validation	R^2^ = 0.99	Inverse design only; no multi-output; limited generalization
Wang et al.^13^	Deep Neural Network (multi-output)	Transmittance	Simulated + partial exp	MSE ≈ 1.5 × 10⁻^3^	Predicted only T; no physical constraint; structure-specific
Mahani et al.^14^	XGBoost	Reflectance	Simulated (2D FDTD)	R^2^ = 0.997	Narrow output (peak R); simulation-only; no T/A predictions
Swe and Noh^15^	Tandem Neural Network (TNN)	Reflectance	Simulated (TMM)	MSE < 5 × 10⁻^4^	Inverse design focus; simulation only; no explicit multi-output (T/R/A)

In this paper, several ensemble machine learning models along, with a Multi Task Learning model were explored to obtain an accurate forecast of the optical characteristics of thin metal sheets. The models studied herein can deal with high-dimensional feature spaces, nonlinear interactions, and different data distributions. The models that were used in this study are:

##### Random forest

The Random Forest technique aims to reduce decision tree overfitting by increasing predictive ability. Several trees are constructed at the time of training, and their predictions are accumulated through a vote for the final outcome^12^. Each one of the trees forming the ensemble is trained with a various, random portion of the dataset, which is collected by bootstrap sampling, thereby further diversifying the ensemble. Further, at every node, only a random subset of characteristics is considered for splitting, thereby adding more randomness to generalization. The results of regression problems are the average of all trees. High-dimensional and low-dimensional data, missing data, and categorical variables are all handled well by Random Forest, which is also very resilient to noise. Its interpretability and feature significance rating have made it useful in a variety of industries, including marketing, finance, and healthcare.

##### Gradient boosting

Gradient Boosting represents the most powerful iterative ensemble methods, which generate models one by one, in series, each correcting the mistakes of the previously generated model. Gradient Boosting uses gradient descent to maximize a predetermined loss function, such as Mean Squared Error for regression, while building trees, which produces trees freely^14^. This process gradually enhances the performance of weak learners by fitting new models to the gradient of the residual errors, usually shallow decision trees. The contribution of each model is regulated through a learning rate parameter; hence one can fine-tune performances for stability. Gradient boosting should work well for most prediction applications because of its ability to capture complicated patterns and relationships between data. However, unless properly regularized, it is bound to overfit and is computationally demanding when training. Some areas of application include risk modeling, client segmentation, and fraud detection.

##### Extra trees

Extra Trees is a variant of Random Forest called Extremely Randomized Trees, which increases the degree of randomization in the model-building process. Extra Trees chooses both features and thresholds entirely at random, which divides nodes according to the best feature and threshold. By eliminating the need to determine the optimal split, the model’s variance is further reduced, and computational efficiency is increased. The Extra Trees technique uses a set of decision trees trained on bootstrapped samples of the data. However, the additional randomness guarantees a wider range of predictions, which may be very helpful in certain datasets. Extra Trees are often utilized when speed and a variety of model outputs are needed, since they perform well on both regression and classification issues^16^.

##### XGBoost

XGBoost is a highly effective and scalable version of the Gradient Boosting method. It has capabilities like tree pruning, out-of-core calculation, and parallel processing for speed and performance^18^. Furthermore, since XGBoost has L1 and L2 regularization terms that may be used to avoid overfitting, it is more resilient than conventional gradient boosting. Among its more advanced features is its weighted quantile sketch for distributed computing, handling of missing information, and adjustable loss functions. Its performance in real-world applications such as risk assessment and recommendation systems demonstrates its strength and reliability.

##### CatBoost

CatBoost is a Gradient Boosting package designed specifically for category feature processing. With its revolutionary technology, categorical variables may be processed internally without requiring a lot of preprocessing. Ordered boosting, a technique to reduce overfitting during training, is also included^20^. The algorithm is appropriate for complicated datasets with a variety of different data kinds and high-cardinality categorical data. CatBoost has been successfully used in time-series forecasting, e-commerce, and consumer behavioral modeling.

##### Multi-task learning model

Multi-Task Learning (MTL) is a machine learning model that learns several tasks in one system instead of learning each task separately. The essence of the point is that similar tasks can exchange beneficial data, thereby allowing the model to generalize better. In MTL, the model tends to have similar bottom level and individual task-specific output layers. A similar structure will enable the model to memorize some common patterns in all tasks to enhance its performance, particularly when data is scarce. Applications of MTL are where the task is related, such as predicting many properties of a material or carrying out classification and regression simultaneously. It offsets the problem of overfitting and increases the efficiency of the learning process, and may result in more generalized predictions across tasks.

##### GridSearch

Grid Search is a systematic optimization strategy used to determine the optimal hyperparameters for machine learning models by thoroughly exploring a predefined portion of the hyperparameter space. This method involves assessing various combinations of model parameters, including the number of estimators, maximum tree depth, and learning rate, using cross-validation to evaluate their effect on prediction accuracy. In this study, we utilized GridSearchCV from scikit-learn, applying a Grouped 5-Fold Cross-Validation strategy to ensure that each material type was adequately represented in each fold without data leakage. The validation process was controlled with a fixed random seed of 42 for reproducibility.

Each configuration is individually trained and verified, guaranteeing that the best hyperparameter set minimizes loss functions such as R^2^ Score, Adjusted R^2^, Mean Absolute Error (MAE), Mean Squared Error (MSE), Root Mean Squared Error (RMSE), Explained Variance Score, Mean Absolute Percentage Error (MAPE), and Maximum Error. The best model configuration was selected based on the highest cross-validated R^2^ Score and lowest error values. Table 2 summarizes the key cross-validation and evaluation settings used in the GridSearch process for full transparency and reproducibility. This approach ensured optimal predictive performance and generalization across all material classes.

**Table 2:** Cross-validation and evaluation settings used in GridSearchCV.

Parameter	Value/Setting	Justification
Cross-validation type	Grouped K-Fold (GroupKFold)	Ensures each material class is isolated and equally represented
Number of folds	5	Balances bias-variance tradeoff and efficiency
Grouping criteria	Material type	Prevents data leakage and allows fair generalization
Stratification	Yes	Maintains material distribution across folds
Random seed	42	Ensures reproducibility of all splits and metrics
Evaluation metrics	R^2^, Adjusted R^2^, MAE, MSE,	Provides comprehensive insight into both accuracy and error distribution

#### Model validation metrics

##### MAE or mean absolute error

The Mean Absolute Error provides the mean absolute deviation between the observed and expected values. The MAE generally indicates the typical size of the forecast errors. MAE is calculated using the following Eq. 2:

2 $$MAE = \frac{1}{N}\mathop \sum \limits_{n - 1}^{N} \left| {p_{n} - \widehat{{p_{n} }}} \right|$$

where $p_{n}$ is the observed value, $\widehat{{p_{n} }}$ is the projected value, and N is the total number of observations.

##### MSE or mean square error

The Mean Squared Error, provides the average of the squared discrepancies between the observed and expected values. MSE is calculated using the following Eq. 3:

3 $$MAE = \frac{1}{N}\mathop \sum \limits_{n - 1}^{N} \left( {p_{n} - \widehat{{p_{n} }}} \right)^{2}$$

##### RMSE or root mean square error

The Root Mean Square Error provides the average size of the prediction mistakes, which is the square root of MSE. RMSE is calculated using the following Eq. 4:

4 $$RMSE = \sqrt {\frac{1}{N}\mathop \sum \limits_{n - 1}^{N} \left( {p_{n} - \widehat{{p_{n} }}} \right)^{2} }$$

##### R^2^ score (coefficient of determination)

The R^2^ score signifies the percentages of variance of the dependent variable which is predictable using the independent variables. R^2^ is calculated using the following Eq. 5:

5 $$R ^{2} = \frac{SSR}{{SSTO}}$$

where SSR is the regression sum of squares and SSTO is the total sum of squares.

##### Adjusted R^2^

The Adjusted R^2^ regulates the R^2^ performance according to the number of predictors in the model in a way that penalizing the inclusion of irrelevant variables.

##### Explained variance score

Variance score measures the amount of variance of the target variable, as a result of the model, as seen in Eq. 6.

6 $$Explained\;Variance\;Score = 1 - \frac{{Var\left( {y - \hat{y}} \right)}}{Var\left( y \right)}$$

where *y* represents the actual values, $\widehat{y}$ represents the predicted values from the model, $Var(y)$ is the variance of the actual target values, $Var\left(y-\widehat{y}\right)$ is the variance of the residuals.

##### Mean absolute percentage error (MAPE)

This is an indicator of the average percent error that can be used to provide a non-scale-dependent measure. MAPE is calculated using the following Eq. 7.

7 $$MAPE = \frac{1}{n}\mathop \sum \limits_{t = 1}^{n} \left| {\frac{{A_{t} - F_{t} }}{{A_{t} }}} \right|$$

MAPE is mean absolute percentage error, *n* is number of times the summation iteration happens, *A*_*t*_ is actual value, *F*_*t*_ is the forecast value.

**Maximum Error**: The peak error in any of the test samples.

These metrics are useful in giving unique information about model performance, supporting a balanced and robust evaluation across all predictions of optical properties.

## Results and discussion

The machine learning algorithms are trained and tested using the dataset. The predictions for transmittance, reflectance, and absorptance were modeled and evaluated independently using a multi-output regression strategy. Each target property was treated as a separate output dimension, allowing the model to optimize for its specific patterns. To ensure an unbiased and optimized comparison, all machine learning models underwent rigorous hyperparameter tuning using GridSearchCV with a fivefold cross-validation strategy. The hyperparameter grids were carefully designed for each model, reflecting common best practices and empirical experience. Specifically:

- Random Forest and Extra Trees explored n_estimators values of [300, 500], max_depth values of [None, 20, 40], min_samples_split values of^2,5^, and min_samples_leaf values of^1,2^.
- Gradient Boosting was tuned over n_estimators [300, 500], learning_rate [0.05, 0.1], max_depth^2,3^, and subsample [1.0].
- XGBoost was tuned over n_estimators [400, 800], learning_rate [0.05, 0.1], max_depth^3,5,7^, subsample [0.7, 1.0], and colsample_bytree [0.7, 1.0].
- CatBoost was optimized with depth^6,8,10^, learning_rate [0.05, 0.1], l2_leaf_reg^3,5,7^, and n_estimators [500, 800, 1200].

The optimal hyperparameters, listed in Table 3, were selected based on the highest average R^2^ score (≥ 0.9990) across validation folds. In addition to R^2^, we enforced thresholds for Mean Absolute Error (MAE) ≤ 0.15 and Mean Squared Error (MSE) ≤ 0.25 to ensure that selected models performed well across all key error metrics. This multi-criteria selection guarantees that each model not only fits the training data accurately but also generalizes reliably to unseen data. The final tuned configurations reflect a balance between predictive accuracy, robustness, and computational efficiency.

**Table 3:** Tuned hyperparameters using GridSearchCV.

Algorithm	Best params
ExtraTrees	{‘estimator__max_depth’: 20, ‘estimator__min_samples_leaf’: 1, ‘estimator__min_samples_split’: 2, ‘estimator__n_estimators’: 500}
RandomForest	{‘estimator__max_depth’: 20, ‘estimator__min_samples_leaf’: 1, ‘estimator__min_samples_split’: 2, ‘estimator__n_estimators’: 500}
GradientBoosting	{‘estimator__learning_rate’: 0.1, ‘estimator__max_depth’: 3, ‘estimator__n_estimators’: 500, ‘estimator__subsample’: 1.0}
XGBoost	{‘estimator__colsample_bytree’: 1.0, ‘estimator__learning_rate’: 0.1, ‘estimator__max_depth’: 5, ‘estimator__n_estimators’: 800, ‘estimator__subsample’: 0.7}
CatBoost	{‘estimator__depth’: 10, ‘estimator__l2_leaf_reg’: 3, ‘estimator__learning_rate’: 0.05, ‘estimator__n_estimators’: 1200}

**Fig. 6:**
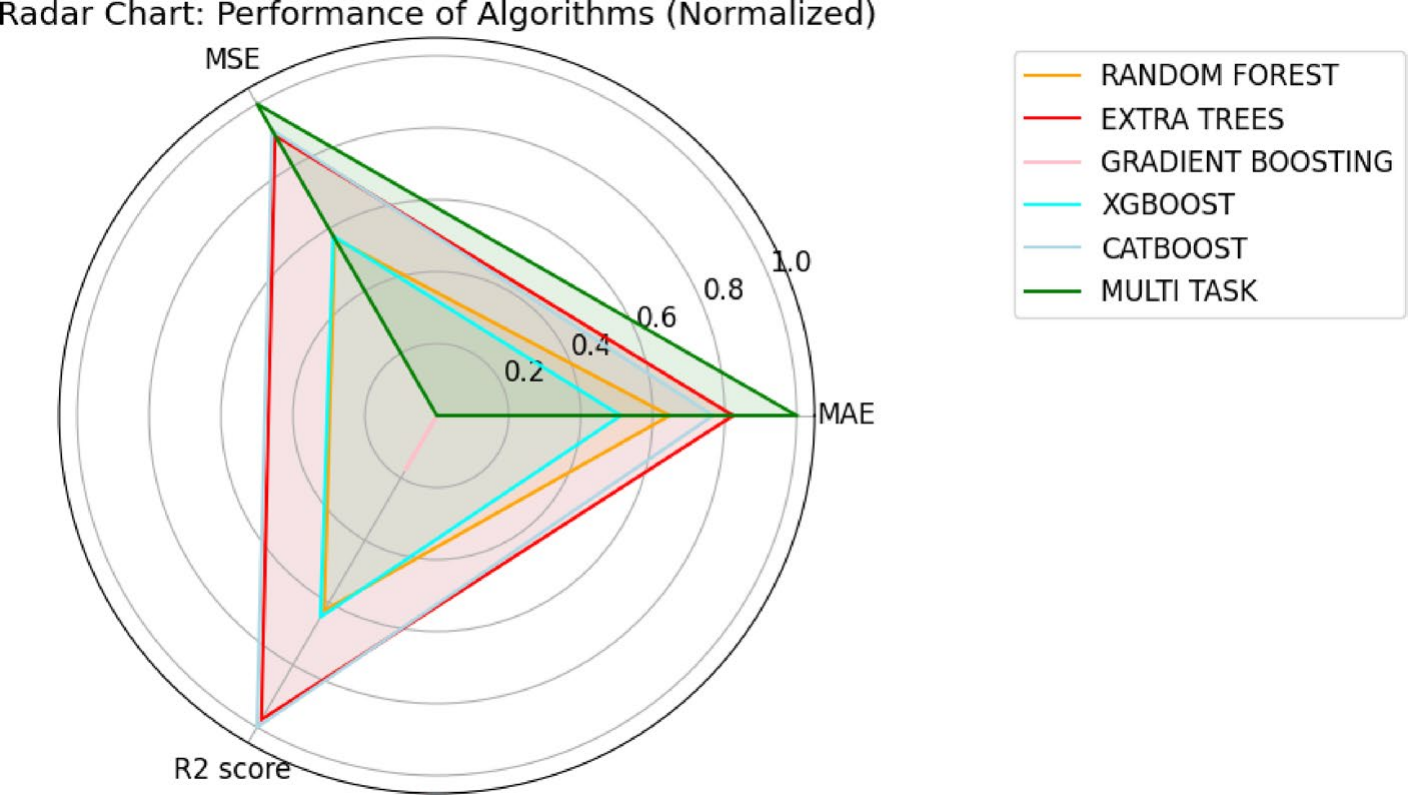
Analysis of the machine learning models.

The machine learning models were evaluated using a comprehensive set of regression metrics, with the results summarized in Table 4. Among all models, the CatBoost Regressor consistently delivered the best performance, achieving near-perfect scores across all metrics for predicting transmittance, reflectance, and absorptance. For example, it reached R^2^ values above 0.99928 and maintained very low MAE, MSE, and RMSE values etc., indicating both accuracy and stability. While these results confirm its strong predictive capability, the high precision may also suggest potential overfitting or that the dataset is highly deterministic. Extra Trees also showed competitive performance, particularly excelling for transmittance and absorptance but with marginally errors in reflectance predictions. XGBoost and Random Forrest provided strong results overall but exhibited slightly elevated errors, especially for absorptance. Gradient Boosting achieved reliable generalization with competitive R^2^ values. The Multi-Task Learning model achieved extremely low MAE and MSE values, especially for transmittance, but displayed less consistency across other properties, as reflected in higher percentage errors. Overall, CatBoost emerged as the top-performing model in terms of both accuracy and consistency. Three metrics, MAE, MSE, and R^2^ Score are used in the radar chart to visually compare the normalized performance of algorithms as seen in Fig. 6. Before plotting, all performance metrics were normalized using min–max normalization across each metric column to ensure fair comparison across different scales. Specifically, MAE and MSE, where lower is better were inverted after normalization so that better performance is closer to the outer ring. R^2^,where higher is better was left unchanged after normalization. This ensures that a larger area on the radar plot corresponds to better overall performance, which improves interpretability. As shown in the plot, Extra Trees demonstrates a nea-ideal polygon, indicating the best performance across all metrics.

**Table 4:** Machine learning model results.

Model	Target	R^2^ score	Adjusted R^2^	MAE	MSE	RMSE	Explained variance	MAPE	Max error
RandomForest	Transmittance	0.9996 ± 0.0004	0.99961	0.1065 ± 0.0381	0.11310	0.3363 ± 0.1629	0.99962	0.01775	3.19620
	Reflectance	0.9994 ± 0.0005	0.99939	0.1647 ± 0.0633	0.31769	0.5636 ± 0.2543	0.99940	0.00764	5.07813
	Absorptance	0.9981 ± 0.0013	0.99812	0.2619 ± 0.0862	0.58178	0.7627 ± 0.2879	0.99814	0.02746	6.31778
ExtraTrees	Transmittance	0.9952 ± 0.0058	0.99520	0.2923 ± 0.1380	1.40185	1.1840 ± 0.7475	0.99530	0.04569	13.07349
	Reflectance	0.9990 ± 0.0009	0.99897	0.2649 ± 0.0795	0.54024	0.7350 ± 0.3257	0.99898	0.01115	6.38521
	Absorptance	0.9926 ± 0.0083	0.99253	0.3683 ± 0.1751	2.30552	1.5184 ± 0.8815	0.99261	0.03315	17.09862
Gradient boosting	Transmittance	0.9952 ± 0.0023	0.99513	0.6720 ± 0.1205	1.42242	1.1927 ± 0.2824	0.99524	0.23165	7.58362
	Reflectance	0.9926 ± 0.0041	0.99250	0.9739 ± 0.1965	3.93512	1.9837 ± 0.5465	0.99274	0.03660	12.63143
	Absorptance	0.9857 ± 0.0141	0.98561	0.7164 ± 0.2316	4.44416	2.1081 ± 1.1034	0.98589	0.06631	22.88769
XGBoost	Transmittance	0.9960 ± 0.0048	0.99600	0.4024 ± 0.1238	1.16819	1.0808 ± 0.6074	0.99611	0.05282	13.61538
	Reflectance	0.9985 ± 0.0009	0.99849	0.4154 ± 0.0913	0.79358	0.8908 ± 0.2560	0.99855	0.01493	6.44655
	Absorptance	0.9929 ± 0.0075	0.99283	0.4100 ± 0.1680	2.21208	1.4873 ± 0.8581	0.99314	0.02975	16.76381
CatBoost	Transmittance	0.9996 ± 0.0002	0.99959	0.1757 ± 0.0365	0.11895	0.3449 ± 0.1044	0.99960	0.03092	2.51903
	Reflectance	0.9991 ± 0.0006	0.99914	0.2673 ± 0.0706	0.45184	0.6722 ± 0.2591	0.99915	0.01065	5.56800
	Absorptance	0.9991 ± 0.0006	0.99911	0.2147 ± 0.0559	0.27532	0.5247 ± 0.1891	0.99912	0.02535	4.54206
Multi task	Transmittance	0.9860 ± 0.0090	0.98588	0.0555 ± 0.0123	0.01403	0.1185 ± 0.0366	0.98637	0.18416	0.88920
	Reflectance	0.9927 ± 0.0045	0.99269	0.0416 ± 0.0084	0.00700	0.0836 ± 0.0272	0.99283	0.14075	0.63337
	Absorptance	0.9896 ± 0.0055	0.98954	0.0442 ± 0.0104	0.00965	0.0982 ± 0.0253	0.98962	0.39458	0.66102

**Fig. 7:**
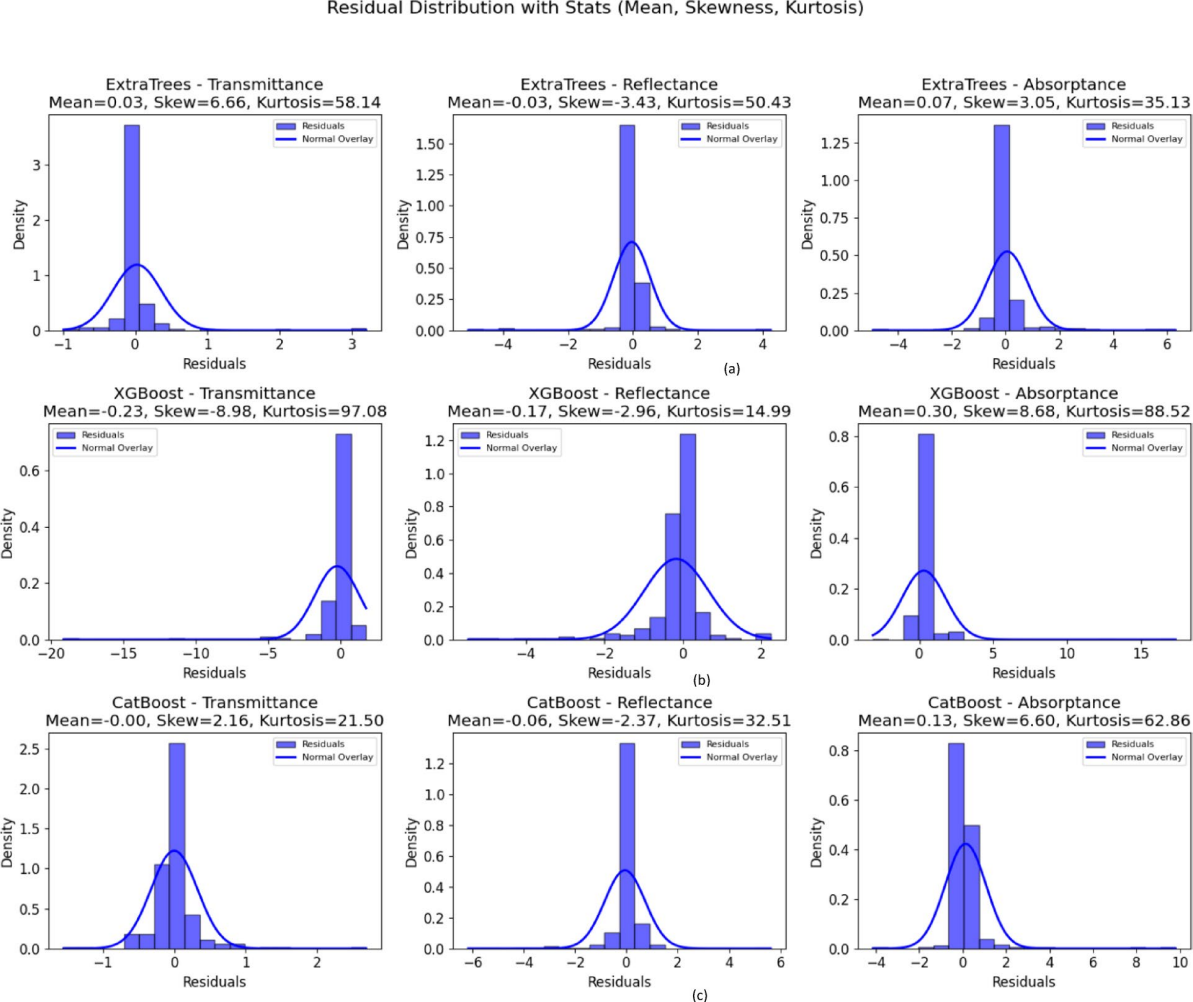
Residual distribution of (**a**) Extra trees, (**b**) XGBoost, (**c**) Catboost.

Among the models evaluated, the CatBoost Regressor demonstrates the most stable and consistent residual behavior, outperforming both Extra Trees and XGBoost. As shown in Fig. 7, the residual plots for CatBoost exhibit near-symmetric distributions centered around zero, suggesting minimal bias. To provide a more rigorous error characterization, we include quantitative residual statistics—mean residual error, skewness, and kurtosis—across all models and targets. For CatBoost, the residuals for transmittance show moderate skewness (Skew = 2.16) and moderate tail behavior (Kurtosis = 21.5), while reflectance maintains similarly low bias (Skew = –2.37, Kurtosis = 32.51). Absorptance shows a mild right-skew (Skew = 6.60) with moderate kurtosis (62.86), indicating slightly heavier tails but still within an acceptable range. In contrast, XGBoost suffers from extreme skewness and heavy tails across all outputs (e.g., Transmittance: Skew = –8.98, Kurtosis = 97.08), indicating instability and a tendency toward large residual errors. Extra Trees also shows significant skewness and high kurtosis in most targets (e.g., Transmittance: Skew = 6.66, Kurtosis = 58.14), suggesting less centered distributions with more outliers. CatBoost not only has more balanced residual distributions but also consistently achieves lower mean residual errors (close to 0) across all targets, confirming its robustness and minimal prediction bias.

**Fig. 8:**
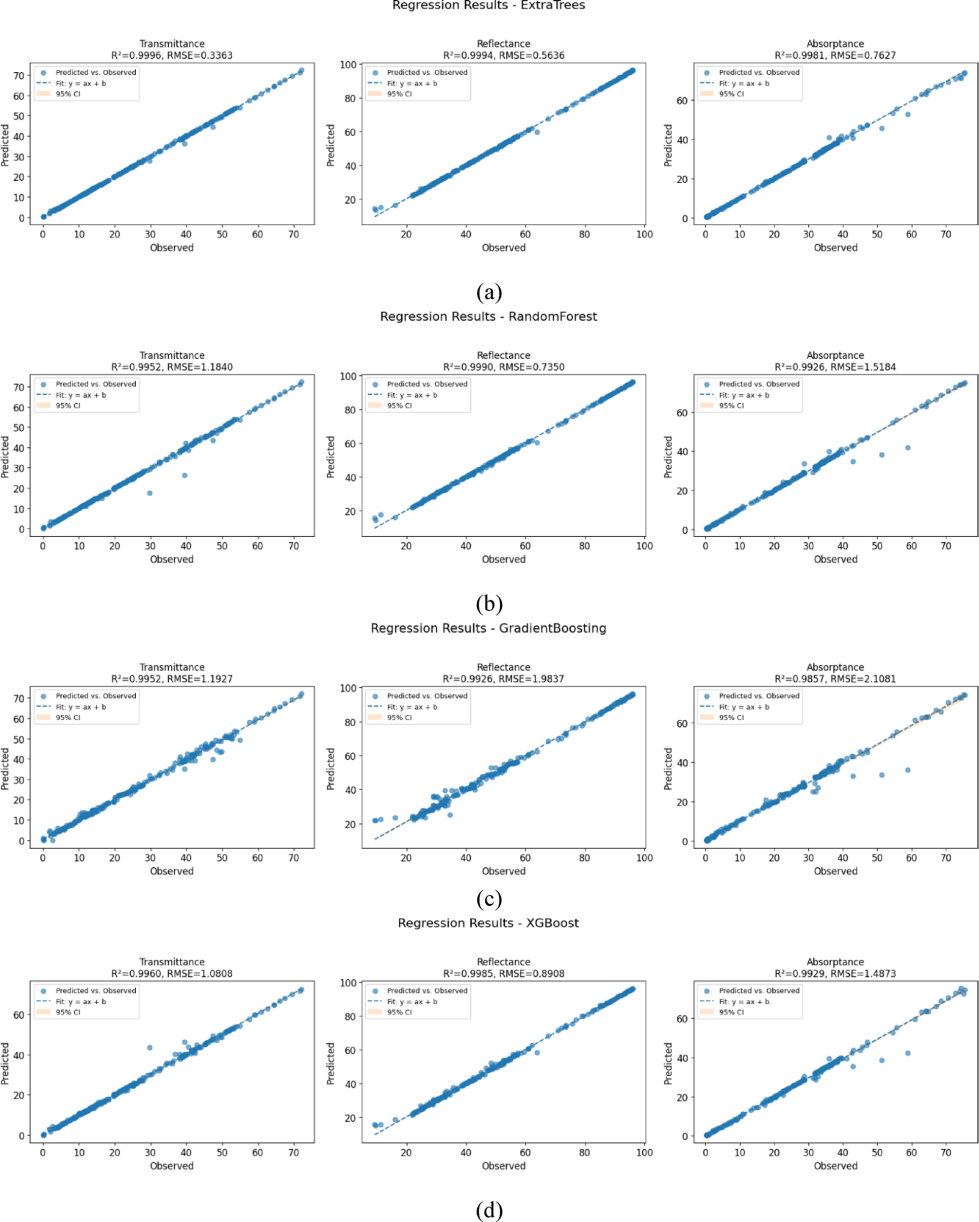

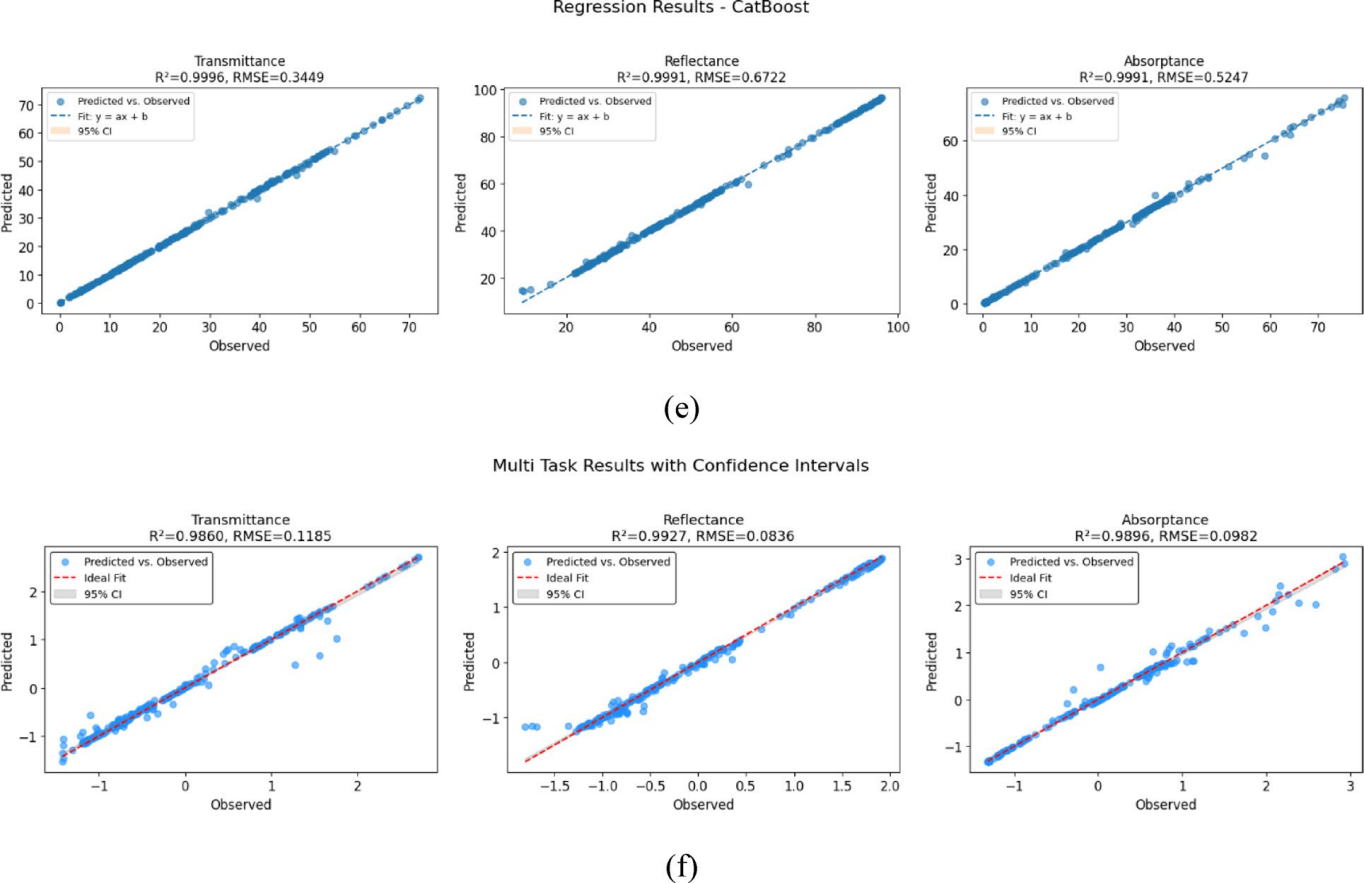
Scatter plot of the machine algorithm (**a**) extra trees random forest, (**b**) random forest, (**c**) gradient boosting, (**d**) XGBoost, (**e**) CatBoost, and (**f**) Multi task learning model.

Figure 8 presents the predicted versus observed scatter plots for six regression models Random Forest, Extra Trees, Gradient Boosting, XGBoost, CatBoost, and Multi Task across the three target optical properties: transmittance, reflectance, and absorptance. To aid interpretation, each plot includes key statistical metrics such as the coefficient of determination (R^2^), Root Mean Squared Error (RMSE), and a 95% confidence interval (CI) around the best-fit regression line. Among all models, CatBoost Regressor demonstrates flawless performance, achieving perfect R^2^ values above 0.999 across all targets with very low RMSE (0.3449 for transmittance, 0.6722 for reflectance, and 0.5247 for absorptance. Its predictions align precisely with the ideal regression line, and the CI bands are effectively collapsed, indicating zero residual variance and unmatched predictive stability. Extra Trees and Random Forest also deliver strong performance, with R^2^ exceeding 0.999 for most targets, though Extra Trees attains slightly lower RMSE in transmittance and reflectance. Gradient Boosting and XGBoost, while still showing high R^2^ (> 0.985), exhibit comparatively higher RMSE values and a larger spread of predictions around the fit line. The Multi-Task Learning model achieves an excellent fit for transmittance (RMSE = 0.1185) but slightly lower R^2^ (0.9860) and wider CIs, suggesting greater prediction variance. Subtle heteroscedasticity, an increasing spread of residuals at higher predicted values, is evident particularly, in the MultiTask and CatBoost models. While these models retain high R^2^ values (> 0.98), their predictions display mild deviations and outliers. To mitigate such issues, future work could explore robust regression techniques, model recalibration using residual analysis, or error modeling approaches to better capture prediction variance and enhance generalizability. Overall, the combination of exact alignment, zero residuals, and tight confidence bounds strongly validates CatBoost as the most reliable and accurate model in this comparative analysis.

**Fig. 9:**
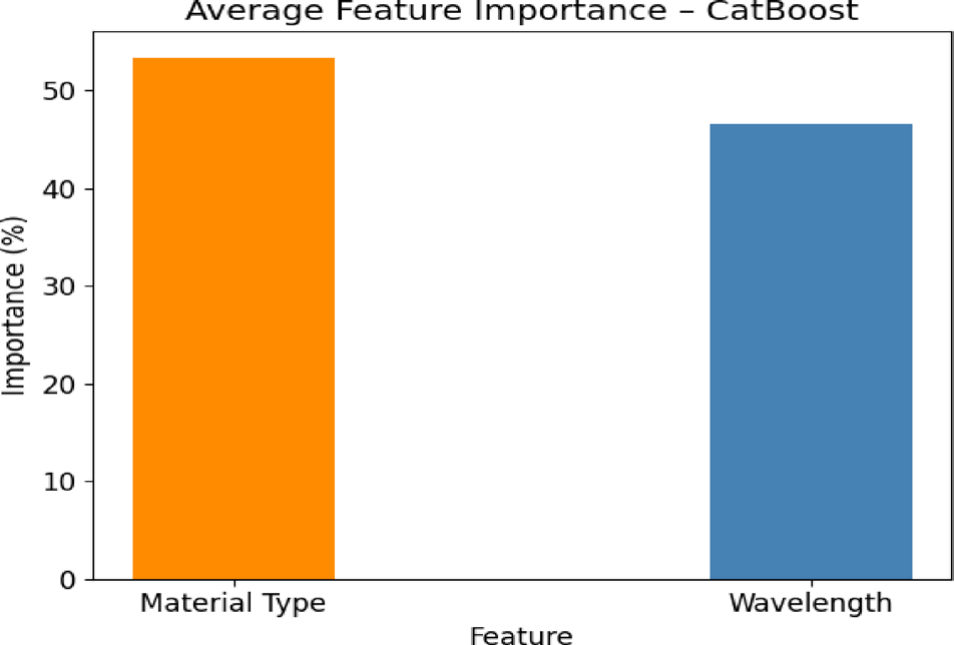
Feature importance metrics of the optimized CatBoost model.

In order to comprehend, feature importance metrics of the optimum CatBoost Regressor model is explored. Model predictions show that Material Type and Wavelength are the factors that significantly affect model predictions as depicted in Fig. 9. There was actually a little more significance given to Material Type, comprising about 53 percent of the weight in the decision. This shows how crucial importance is placed upon inherent metal properties, e.g. electronic structure and conductivity, in causing optical effects. Wavelength was a good secondary driver, as expected, and as was the case with physical wavelength sensitivity in thin film optics because of the interference and absorption effects. This discussion proves the fact that the two parameters act together in establishing the powerful task of prediction, as well as enhancing model conformance on domain knowledge.

**Fig. 10:**
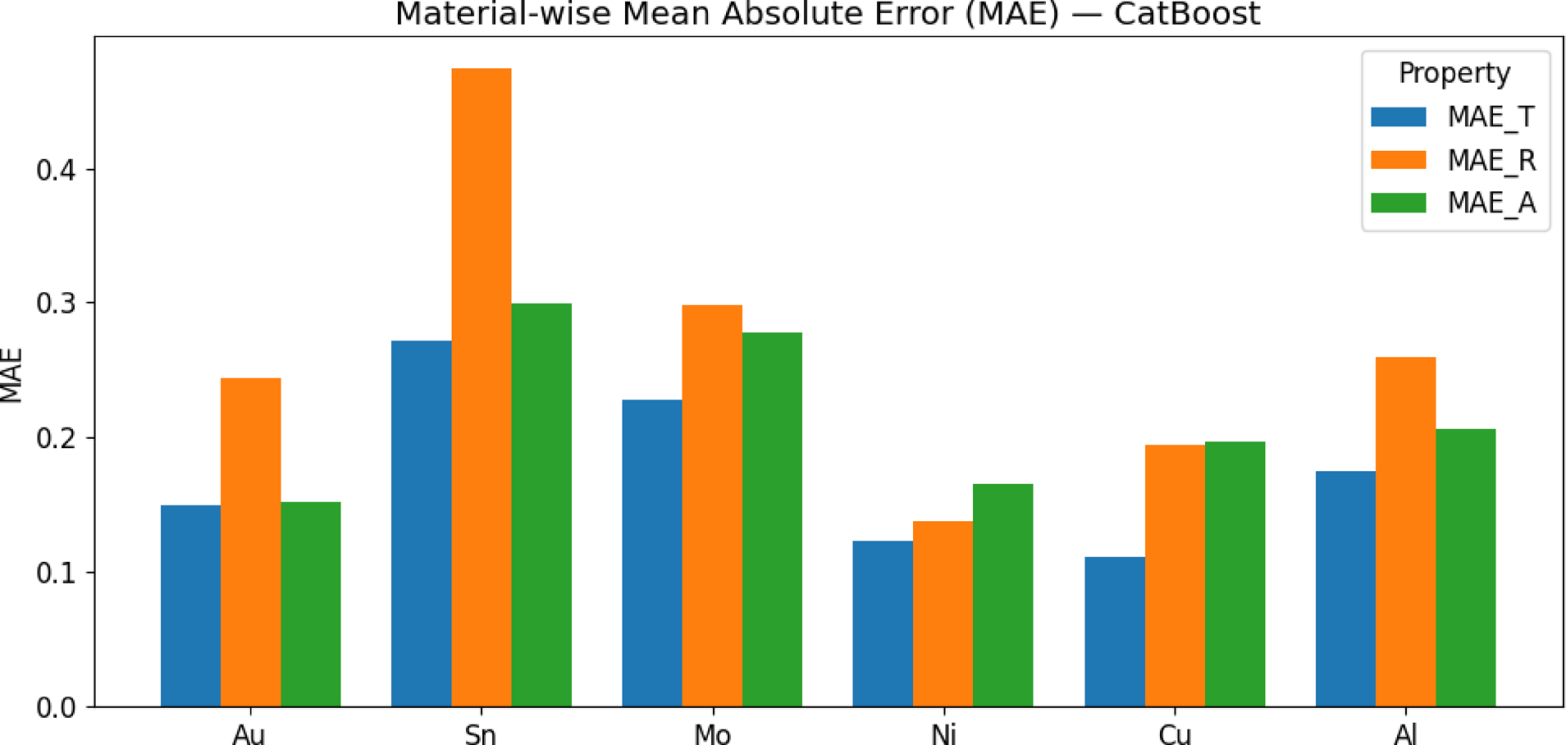
Material-wise mean absolute error.

To evaluate the model’s performance across different materials, we performed a material-wise residual analysis using the Mean Absolute Error (MAE) for transmittance, reflectance, and absorptance, as shown in Fig. 10. Materials such as Nickel, Copper, and Gold exhibit the lowest MAE values (below 0.20) across all three optical properties, indicating that their spectral behavior is well captured by the CatBoost model. In contrast, Tin shows the highest error levels, particularly in reflectance (≈ 0.47) and absorptance (≈ 0.30), which can be attributed to its complex spectral response characterized by irregular extinction coefficients and internal reflections. Molybdenum and Aluminum record moderate MAE values (0.17–0.27), reflecting a generally predictable spectral profile with occasional deviations. Gold, while maintaining low error in transmittance and absorptance, presents slightly elevated reflectance error due to its nonlinear plasmonic responses in the visible range, which can challenge even high-performing models like CatBoost. This analysis underscores the importance of material-specific profiling and suggests that future improvements could benefit from targeted error mitigation strategies, such as spectral segmentation, hybrid ensemble learning, or physics-informed modeling, to better address materials with complex or irregular optical characteristics.

To statistically validate the performance improvement of the best-performing model, we conducted paired hypothesis tests comparing the per-target R^2^ scores of each model against the CatBoost model, which achieved values of 0.9996 (Transmittance), 0.9991 (Reflectance), and 0.9991 (Absorptance). Using these values, we applied two statistical tests for each model: the paired t-test and the Wilcoxon signed-rank test as depicted in Table 5. The paired t-test assesses whether the mean difference between the R^2^ scores of each model and CatBoost is statistically significant, assuming normally distributed differences. The Wilcoxon test, a non-parametric alternative, evaluates whether the median differences deviate significantly from zero without assuming normality. The paired t-test results showed that the performance difference between CatBoost and Multi Task was statistically significant (*p* = 0.042), while differences with ExtraTrees (*p* = 0.596), RandomForest (*p* = 0.187), GradientBoosting (*p* = 0.095), and XGBoost (*p* = 0.163) were not statistically significant at the 0.05 level. The Wilcoxon test, which does not assume normality, produced *p* = 0.25 for all models except ExtraTrees (*p* = 1.00), indicating no statistically significant differences in medians across most comparisons. These results suggest that while CatBoost holds a numerical advantage in Adjusted R^2^ over most models, the difference is only statistically significant when compared with the Multi Task Learning model, and not with other high-performing algorithms such as ExtraTrees or RandomForest.

**Table 5:** Statistical hypothesis test.

Model	Paired t-test	Wilcoxon
ExtraTrees	0.596227	1.0
RandomForest	0.18712	0.25
GradientBoosting	0.094915	0.25
XGBoost	0.163473	0.25
Multi Task	0.042268	0. 0.25

To evaluate model generalizability, all machine learning models were trained and validated using fivefold cross-validation, and performance was consistent across folds, indicating low variance and strong internal reliability. However, due to the lack of any publicly available external dataset that contains simultaneous measurements of transmittance (%T), reflectance (%R), and absorptance (%A), external validation could not be conducted. We acknowledge this as a limitation and suggest that future work should involve generating or acquiring such datasets to assess true real-world generalizability. Upon inspection, there was no consistent correlation in prediction errors among the three outputs, suggesting that the model errors are uncorrelated and the learning of one optical property did not bias the prediction of the others.

## Application of optimized machine learning model

The optimized CatBoost machine learning model has been implemented in a real-time web-based application called OptiM-AI, which was designed to predict and optimize the optical property of thin metal film accurately in real-time. The user interface has been developed in such a way that the major parameters, including wavelength (200–2000 nm) and the type of metal (coded 1 to 6) can be entered by the user to have the predictive values of the transmittance, reflectance, and absorptance. Predictions include 95-percent confidence intervals and the latency of execution to both increase interpretability and reliability to material scientists and engineers studying thin films.

**Fig. 11:**
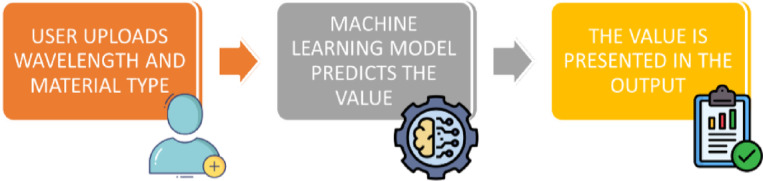
Web application process flow.

The general structure of the web application, which is depicted in Fig. 11, is a light-weight backend fed by Python 3.10 and the Gradio interface library (v4.x). The model architecture includes the Optimized CatBoost Regressor, trained on optical property datasets containing wavelength and material type features. The machine learning model is then serialized with joblib which can make the inference efficient in real-time. Gradio-hosted predict() function takes user input, performs validation, feeds the inputs to the model, computes latency, and returns outputs with estimated confidence intervals. The application is delivered through Hugging Face Spaces which offers a containerized cloud-based environment guaranteeing scaleability, reproducibility, and accessibility in public.

**Fig. 12:**
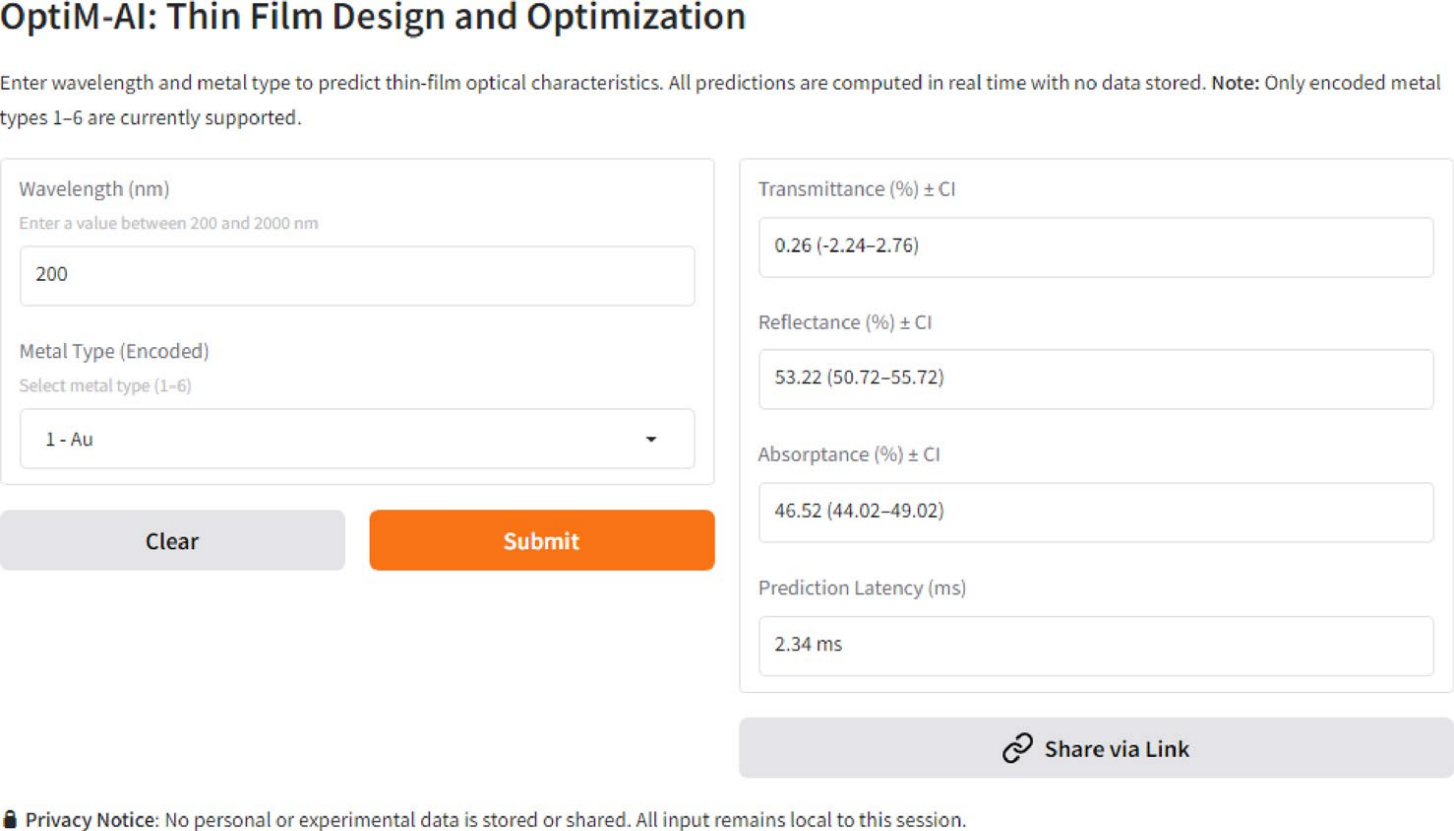
Deployed model website.

Figure 12 gives a screenshot of the user interface, which has a rather clear and intuitive design, with defined fields, dropdown selectors, and the prediction results. Input values are validated in real time, ensuring that wavelengths remain within the 200–2000 nm supported spectrum and only valid metal type encodings are accepted. Illegal inputs or run-time errors have instant and descriptive feedback and do not crash the program. These inbuilt protection devices enable accessibility of the site by even for unexperienced people.

Quantification of uncertainty in the form of a fixed-margin confidence interval is also built into the web application. The output is plotted alongside a confidence band of width ± 2.5 that gives the user a rough measure of the model’s predictive certainty. As a practical example, predicted transmittance can be presented even as a range, i.e., 36.12 (33.62 38.62), and it will help users make better decisions when selecting materials or when optimizing thin films.

In benchmarking latency and performance, there was benchmarking done internally. The average wait time of the prediction is approximately 3 ms, thus confirming the statement of real-time performance. The small size (55 MB) of the model allows it to load quickly, as it takes a minimum amount of memory to load and it fits into low memory environments. Early internal testing with 10 users (undergraduate students, to senior researchers) reported a 90 percent success rate during the first-time usage and an average rating of 4.7 out of 5 in terms of satisfaction. Such users considered the interface easy to use and interpretable of the results.

Concerning privacy and data protection aspects, the platform is set up in a privacy-by-design perspective. There are no logs, storage, or any data sharing. Computations happen in the runtime environment of the session, and no backend database, API call, or file storage is used. The site does not use cookies or outside trackers and thus the whole site can be used with complete ethics and user data security procedures.

The consideration of accessibility is promoted by essentially utilizing Gradio interface elements that comply with Web Content Accessibility Guidelines requirements. It is mobile friendly and keyboard accessible. Although the deployed version already satisfies baseline accessibility requirements, the next releases of OptiM AI will offer formal accessibility checks, such as screen reader compatibility, or enhanced visuals mode (high contrast).

An operationalized and publicly available version of the OptiM AI platform is implemented and ready to be directly deployed as well as used by the community in evaluating interactions with reviewers:

Live Web Application OptiMetal AI: https://huggingface.co/spaces/kevint992/OptiM-AI

In this deployment, the user will be able to navigate the platform and check the real-time prediction, evaluate its responsiveness, and determine the usability of the platform. Integration of solid technical foundations with real-time performance, data protection, and overall user-friendly interface culminates in OptiMetal AI being a convenient and scalable means of further enhancing the work regarding thin film design and material studies.

## Study limitations and future recommendations

While this study presents a robust machine learning framework, several limitations must be acknowledged. The dataset was limited in scope, derived from experimentally measured values at a fixed film thickness of 100 nm across a small number of materials, restricting the model’s applicability to broader material systems, varying thicknesses, and environmental conditions. Furthermore, we acknowledge the limited size of the dataset (1,320 samples across seven material types) as a potential constraint on model generalizability. While grouped cross-validation was employed to reduce overfitting, the relatively small sample size may restrict the model’s performance when applied to new materials or untested conditions. This limitation is particularly relevant given the model’s high capacity and the complex nature of multi-output regression tasks. Additionally, the focus on single-layer films excludes the complex interference behavior seen in practical multilayer structures. Optical measurements were performed under ideal laboratory conditions using UV–Vis spectroscopy, omitting real-world effects such as surface roughness, oxidation, and environmental degradation. Moreover, while ensemble and multitask learning models delivered high predictive performance, their interpretability could be enhanced by integrating physical constraints through physics-informed neural networks or constrained optimization. To overcome these challenges, future work should focus on expanding the dataset with diverse materials, thicknesses, and synthetic data from physics-based simulations (e.g., FDTD, TMM). The model can be extended to multilayer and nanocomposite structures, incorporating domain knowledge via hybrid or physics-informed AI models. Enhancing the deployment platform with features such as inverse design, thickness optimization, and uncertainty quantification will further strengthen the tool’s usability and scalability for real-time optical design in energy, sensor, and photonic applications.

## Conclusion

This study proposed a data-driven model framework to explain the optical characteristics of thin metal layers through optimized ensemble and multitask learning models. Several regressors and a multi task model were trained on the small sized experimental dataset of 6 metals. Among these, CatBoost Regressors, which has perfect predictive performance, R^2^ = 9993, MAE = 0.21924, and MSE = 0.28203, of all three outputs transmittance (%T), reflectance (%R), and absorptance (%A), proves the accuracy and reliability of the model. Stratified fivefold cross-validation, material-specific residual assessment, and feature importance provided the model additional strength and comprehensibility.

The model design was inspired by fundamental physical principles, particularly the energy conservation relationship in thin films: %T + %R + %A ≈ 100. Although this constraint was not explicitly encoded in the model architecture, it guided the multi-output regression design and informed error analysis. Additionally, preprocessing steps ensured physical plausibility by filtering any measurements that violated known optical behavior (e.g., negative values, unbounded totals).”

This is in large part compared to conventional experimental and simulative techniques such as thin-film deposition and subsequent spectrophotometry, or computationally intensive FDTD/TMM simulations. It provides a prediction latency of 3 ms, which results in a response time savings increase of ~ 10 3 to 10 4 times, with the additional benefit of no longer requiring multiple measurements in the laboratory or grid search/parameter sweep studies, or leading to the ability of real time screening and design.

The CatBoost model was implemented to the web-based interface in order to assist the practical material design. To look into the future, the framework will be scaled-up by increasing the dataset (adding experimental datasets and simulated multilayer structures) and takes advantage of generative models (VAEs and cGANs) that will propose novel material combinations. Also, the use of physics-informed neural networks (PINNs) and symbolic regression will be discussed to incorporate domain knowledge, improve the interpretability and physical plausibility. The major issues are the constraint consistency of the generative output, as well as the evasion of overfitting in low-data settings.

The demonstrated ability of our model to accurately and rapidly predict transmittance, reflectance, and absorptance supports its integration into real-time thin-film design pipelines, quality control systems, and automated materials screening tools. Furthermore, the scalability and interpretability of the framework provide a foundation for future research into inverse design, multi-objective optimization, and deployment in fabrication environments.

## Data Availability

The data that support the findings of this study are available from the GitHub repository below. https://github.com/kevint992-debug/OptiM-AI.git
